# Cancer cachexia as an immunometabolic syndrome: targeting the immunometabolic interface with traditional Chinese Medicine monomers and formulas

**DOI:** 10.3389/fimmu.2026.1934109

**Published:** 2026-09-22

**Authors:** Baoyi Ni, Shuhang Wang, Xi Zhang, Zhu Liu, Weiming Wang, Huan He, Jia Wang

**Affiliations:** 1 Mudanjiang Medical University, Mudanjiang, China; 2 Mudanjiang City Hospital of TCM, Mudanjiang, China; 3 Xuyi County People’s Hospital, Huai’an, Jiangsu, China; 4 Hongqi Hospital Affiliated to Mudanjiang Medical University, Mudanjiang, China

**Keywords:** cancer cachexia, immune checkpoint inhibitors, immunometabolism, traditional Chinese medicine, tumor microenvironment

## Abstract

Cancer cachexia is conventionally viewed as muscle wasting but is increasingly recognized as a tumor-driven immunometabolic syndrome. This systemic disorder is characterized by a vicious cycle of metabolic dysregulation, systemic inflammation, and immune evasion, severely compromising the efficacy of immune checkpoint inhibitors (ICIs). Conventional single-target therapies have largely failed to disrupt this redundant pathological network. Here, we comprehensively review the therapeutic potential of Traditional Chinese Medicine (TCM) as multi-target network modulators acting at the immunometabolic interface. At the micro-level, TCM-derived monomers (such as curcumin, andrographolide, and epigallocatechin gallate (EGCG)) bidirectionally target this interface by concurrently suppressing muscle catabolism (via ubiquitin-proteasome and ferroptosis pathways) and reprogramming the immunosuppressive tumor immune microenvironment (TIME) (downregulating PD-L1 and inhibiting MDSCs/Tregs). At the macro-level, chromatographically characterized multi-herb formulas offer systemic recalibration via the gut-muscle-immune axis, utilizing microbial metabolites to restore intestinal barrier integrity, curb systemic inflammation, and epigenetically reinvigorate exhausted CD8+ T cells. Clinically, integrating TCM with ICIs presents a promising synergistic strategy to restore patient eligibility, optimize immunotherapeutic responses, and mitigate treatment-related adverse events. This review provides a mechanistic framework for positioning TCM as a potential systemic adjuvant to break the cachexia-immunosuppression loop in modern oncology.

## 1 Introduction

Cancer cachexia has conventionally been defined as progressive loss of skeletal muscle mass, with or without fat loss, that, if uncontrolled, advances to irreversible functional impairment in the refractory stage. More recent evidence redefines cachexia as a tumor-driven immunometabolic syndrome—a systemic condition where profound metabolic dysregulation (e.g., hypercatabolism) is inextricably linked to, and driven by, aberrant host immune responses and chronic inflammation (1, 2). In advanced malignancies, the systemic inflammatory milieu—sustained by cytokines including IL-6, TNF-α, and TGF-β—simultaneously activates proteolysis and lipolysis and shapes an immunosuppressive TIME (3). This immunometabolic crosstalk—a complex bidirectional communication network where microbial, inflammatory, and metabolic signals are exchanged across distant organs—places patients in a self-reinforcing state: progressive tissue wasting fuels immune exhaustion (CD8+ T cell depletion, M2 macrophage polarization, myeloid-derived suppressor cell [MDSC] expansion), which in turn accelerates further muscle and fat loss. Clinically, this interplay markedly reduces tolerance to anticancer therapy, and cachexia independently predicts poor response to ICIs and shortened overall survival (OS) (4, 5).

Despite this unmet need, current pharmacologic interventions, which rely predominantly on single-target approaches, face structural limitations in disrupting this redundant, multifactorial network. Megestrol acetate, an appetite stimulant, increases adipose tissue without preserving skeletal muscle and carries thromboembolic risk (6). The ghrelin receptor agonist anamorelin improves lean mass but has not been shown to enhance physical function such as handgrip strength (7). Glucocorticoids, frequently used for short-term symptom palliation, exemplify a therapeutic contradiction: they transiently improve appetite but directly activate muscle-specific ubiquitin ligases (MuRF1, Atrogin-1), thereby accelerating proximal myopathy and sarcopenia (8). In the immunotherapy setting, systemic corticosteroids further reshape the TIME by suppressing cytotoxic CD8+ T cells and expanding regulatory T cells (Tregs) and MDSCs, contributing to ICI resistance and worse survival (9, 10). Anti-TNF-α biologics have likewise failed to reverse the cachectic phenotype in clinical trials, underscoring the insufficiency of linear, single-node blockade against a complex immunometabolic system.

In this context, TCM represents a distinct therapeutic approach characterized by multi-component, multi-target regulation. Rather than inhibiting single nodes, TCM monomers and botanical formulas function as network modulators. Preclinical and emerging clinical data indicate that TCM can concurrently suppress proteasomal degradation, attenuate systemic inflammation, and reprogram the immunosuppressive TIME—reversing tumor-associated macrophage M2 polarization toward an M1 phenotype, alleviating CD8+ T cell exhaustion, and reducing MDSC accumulation, in part through the gut-microbiota-metabolite axis (11, 12). Emerging evidence further suggests that TCM interventions extend to additional pathological processes, including microbiota-driven gut-muscle-immunometabolic and ferroptosis-dependent myocyte depletion, which are critically examined in this review. By acting at the interface of immune and metabolic dysregulation, TCM offers a strategy to disrupt the cachexia-immune evasion cycle and restore immunological competence.

This review systematically evaluates the molecular circuits that connect systemic metabolic dysregulation with immune subversion, and assesses the therapeutic effects of TCM monomers and formulas at this interface. Two conceptual frameworks are examined in detail: the gut-muscle-immune axis as a basis for formula-driven systemic recalibration, and ferroptosis inhibition as a mechanism for monomer-mediated muscle protection. Throughout this review, cachexia is examined as an immunological disorder as much as a metabolic one. The translational readiness of TCM as a synergistic adjuvant in the immunotherapy era is critically appraised.

### 1.1 Literature search strategy and scope

It should be noted that this article is structured as a non-systematic narrative review. To identify relevant literature, comprehensive searches were conducted in PubMed and Web of Science databases for articles published in English from their inception to recent years. The primary search queries included combinations of terms such as “cancer cachexia”, “immunometabolism”, “tumor immune microenvironment”, “traditional Chinese medicine”, “TCM-derived monomers”, and “multi-herb formulas”. Preclinical studies, clinical trials, and highly cited mechanistic papers that explicitly address the intersection of cachexia, immunity, and TCM pharmacology were prioritized for inclusion. While we aim to provide a broad perspective on the network-modulating potential of TCM, the selection of representative compounds and formulas serves illustrative purposes rather than an exhaustive systematic evaluation.

## 2 The immunometabolic in cancer cachexia

Recent studies have established that cancer cachexia extends beyond nutritional deficiency or isolated muscle atrophy and instead represents a tumor-driven, multi-organ immunometabolic syndrome (1, 2). This conceptual shift is reinforced by recent work framing cachexia as a systemic consequence of progressive, unresolved disease, in which distinct inflammatory, metabolic, and neuro-modulatory drivers converge on advanced tissue wasting (13). Systemic metabolic reprogramming and tissue wasting reshape the TIME, which in turn amplifies distant organ catabolism through a self-reinforcing cycle. The immune system is a central mediator of this wasting process, in which redundant inflammatory cascades, neuro-immune circuits, and altered nutrient availability converge (3). The progressive loss of whole-body homeostasis is therefore linked to marked immune exhaustion, facilitating immune evasion and accelerating disease progression (**Figure 1**).

**Figure 1:**
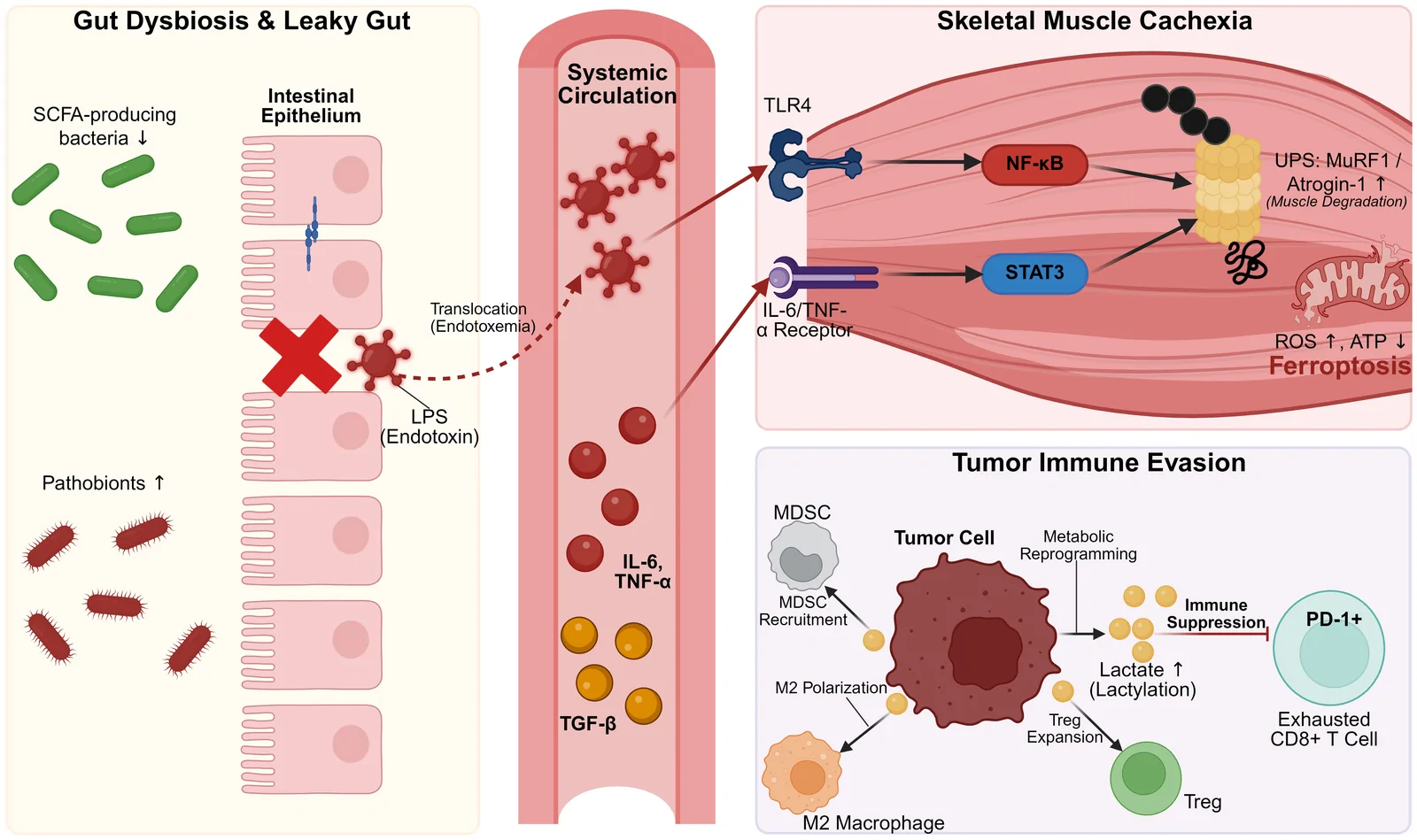
Pathological crosstalk within the gut-muscle-immune axis during advanced malignancy (Created with BioRender.com. Publication license agreement number: FJ2A2ZQKM6. Citation: Jia Wang (2026). BioRender.com/yyyyca5). Intestinal dysbiosis and a compromised barrier (“leaky gut”) allow the systemic translocation of endotoxins (LPS), initiating a whole-body inflammatory cascade (IL-6, TNF-α, TGF-β). In skeletal muscle, these systemic signals activate the TLR4/NF-κB pathway to upregulate the UPS (MuRF1/Atrogin-1) for muscle degradation, while concurrent ROS accumulation and ATP depletion trigger ferroptosis. Simultaneously, tumor-derived lactate drives metabolic reprogramming in the TIME, leading to CD8+ T cell exhaustion (PD-1+) and the recruitment of immunosuppressive cells, including M2 macrophages, Tregs, and MDSCs.

### 2.1 Organ-level crosstalk

#### 2.1.1 The adipose-immune axis: lipotoxicity and macrophage polarization

Adipose tissue wasting often precedes skeletal muscle atrophy and serves as a hub for the generation of immunomodulatory lipid mediators. Cachexigenic tumors drive extensive white adipose tissue (WAT) lipolysis and browning through pro-inflammatory cytokines (e.g., IL-6, TNF-α) and sympathetic neuro-immune circuits. Cachexia induces type 2 immunity in WAT, polarizing macrophages toward an M2 phenotype; this creates a neuroprotective environment for sympathetic neurons that further amplifies β-adrenergic-driven WAT browning and energy expenditure (14).

This multi-organ perspective is further supported by recent reviews that conceptualize cancer cachexia as a syndrome of inter-organ signal communication, in which tumor- and host-derived factors—including inflammatory cytokines, circulating proteins, and metabolites—mediate complex crosstalk between distant organs (15). Such frameworks underscore the need to understand cachexia as a systemic ecosystem rather than a single-organ disease.

The resulting increase in free fatty acids (FFAs) is taken up by tumor-associated macrophages (TAMs) via CD36 and FATP1 transporters. Intracellularly, this lipid influx shifts TAM metabolism from glycolysis toward fatty acid oxidation (FAO) and oxidative phosphorylation, driving STAT6 activation and promoting an immunosuppressive M2 phenotype (16, 17). In parallel, saturated fatty acids (e.g., palmitic acid) and oxidized phospholipids act as damage-associated molecular patterns (DAMPs). These lipid DAMPs engage the TLR4/MD-2 complex on myeloid cells, activating MyD88/NF-κB and NLRP3 inflammasome pathways. This generates a spatially compartmentalized pattern: sustained FAO within the TIME enforces local immunosuppression, whereas systemic lipid-TLR4 engagement sustains a release of IL-6 and IL-1β that perpetuates peripheral tissue catabolism (18). In addition, cancer-associated adipocytes undergo FGF21-driven lipolysis, releasing lipids that induce mitochondrial dysfunction and lipid peroxidation in CD8+ T cells, thereby contributing to terminal T cell exhaustion and immunotherapy resistance (19). Adipose tissue wasting thus reconfigures the immunological landscape by simultaneously enforcing local M2 dominance, driving systemic inflammatory cytokine release, and imposing lipid peroxidation-driven T cell dysfunction. Beyond cytokine-driven macrophage polarization, recent evidence has established direct tumor-macrophage crosstalk as a driver of muscle wasting. Liu et al. demonstrated that tumor cells recruit and reprogram macrophages via the CCL2/CCR2 axis, facilitating non-autonomous TWEAK secretion that directly induces skeletal muscle atrophy (20). This direct “tumor-immune-muscle” communication provides a mechanistic basis for TCM interventions that target macrophage polarization (see Section 3.2).

#### 2.1.2 The muscle-immune axis: myokines, thymic involution, and immune senescence

Skeletal muscle functions as an immunoregulatory endocrine organ, and its progressive atrophy initiates a marked state of immune senescence. Depletion of muscle-derived IL-15, a cytokine required for NK cell survival and cytolytic activity, as well as for homeostatic survival of memory CD8+ T cells (via Bcl-2 maintenance), directly contributes to T cell and NK cell loss during cachexia (21).

Cachectic muscle and tumors overexpress the TGF-β superfamily members myostatin (GDF-8) and activin A. In addition to activating SMAD2/3-mediated muscle proteolysis (e.g., Atrogin-1, MuRF1), the myostatin-activin signaling network upregulates immune checkpoint molecules (PD-L1, CTLA-4, TIM-3) and promotes Treg expansion, linking muscle catabolism with local immune tolerance (22).

Beyond these peripheral effects, cachexia-associated muscle wasting also affects central lymphoid organs. Structural thymic involution disrupts the maturation of thymic medullary fibroblasts (mFbs), impairing tissue-restricted antigen presentation and T cell negative selection (23). Concurrently, polymorphonuclear MDSCs (PMN-MDSCs) infiltrate skeletal muscle and secrete activin A, directly driving local wasting. This establishes a feedforward loop in which immune suppression and muscle catabolism act as mutually reinforcing processes (24).

#### 2.1.3 Tumor-derived extracellular vesicles: remote mediators of catabolism and immune evasion

Tumor-derived extracellular vesicles (EVs) serve as intercellular messengers that coordinate systemic muscle wasting with TIME immunosuppression. EV biogenesis is frequently regulated by the STAT3 pathway, and EVs carry shared molecular cargoes—including DAMPs (HSP70, HSP90, HMGB1), mitochondrial DNA (mtDNA), and microRNAs—that exert dual effects on muscle and immune cells (25, 26).

Surface-bound HSP70 and HSP90 on EVs act as a central mechanistic node. In skeletal muscle, these chaperones bind TLR4 and trigger p38 MAPK, simultaneously activating the ubiquitin-proteasome and autophagy-lysosome pathways. Within the TIME, exosomal HSP70 engages TLR2/MyD88 on myeloid cells, leading to STAT3 phosphorylation, MDSC expansion, and autocrine IL-6 production (27). Anti-tumor immune pressure, such as CD8+ T cell cytotoxicity, can activate the tumor-intrinsic NLRP3 inflammasome, accelerating HSP70-EV release. This pathway recruits PMN-MDSCs to pre-metastatic niches and provides a mechanistic basis for hyperprogression during immune checkpoint blockade (28).

EVs also transfer mtDNA into recipient macrophages, thereby activating the cGAS-STING pathway. While acute cGAS-STING signaling is immunostimulatory, chronic exposure to EV-mtDNA triggers NF-κB-mediated M2 polarization, PD-L1 upregulation, and MDSC recruitment (29). In addition, exosomal microRNAs (e.g., miR-21, miR-29a) act through TLR7/8 to induce muscle proteolysis and simultaneously suppress PTEN in immune cells, expanding the MDSC population. These three cargo categories—chaperones, mitochondrial DNA, and microRNAs—operate through a common principle: tumor EVs utilize a single intercellular delivery system to engage distinct receptor pathways in muscle and immune cells, ensuring that systemic catabolism and immunosuppression advance in parallel.

Beyond organ-specific signaling, recent comprehensive reviews have emphasized that cancer cachexia arises from systemic metabolic remodeling that reciprocally regulates inflammatory responses in the tumor microenvironment (30), and that understanding cachexia requires a multi-organ perspective encompassing metabolic, neurological, and immunological abnormalities across multiple organ systems (31). These integrated perspectives provide a conceptual foundation for the network-modulating TCM strategies discussed in subsequent sections.

### 2.2 Cellular-level crosstalk: the metabolic-immune interface

At the cellular level, the cachectic TIME functions as a metabolic arena in which competition for limited nutrients and an influx of systemic stress hormones collectively impair anti-tumor immunity (32–34). This cellular crosstalk is characterized by a fundamental metabolic asymmetry: effector T cells are metabolically constrained and neuroendocrinologically suppressed, whereas immunosuppressive cells exploit these conditions to gain a survival advantage.

#### 2.2.1 Nutritional deprivation and CD8+ T cell dysfunction

Metabolic competition for glucose and glutamine between highly proliferative tumor cells and CD8+ T cells is a central mechanism of immune evasion. Tumor cells, driven by the Warburg effect, consume large amounts of local glucose, directly restricting the availability of this substrate required for T cell glycolysis. Glucose deprivation suppresses mTORC1 signaling and traps the glycolytic enzyme GAPDH, which subsequently binds to IFN-γ mRNA and blocks its translation, thereby reducing effector cytokine production (35). Furthermore, lactic acid—the abundant byproduct of tumor glycolysis—acidifies the TIME and binds to GLUT10 on CD8+ T cells, directly disrupting their glucose uptake (36).

Parallel to glucose limitation, tumor cells deplete extracellular glutamine. This glutamine deprivation impairs the folliculin-transcription factor EB (FLCN-TFEB) signaling axis in conventional type 1 dendritic cells (cDC1), preventing effective cross-presentation and CD8+ T cell priming (37). Within the TIME, metabolic stress also leads to the accumulation of reactive aldehydes (e.g., acrolein) from lipid peroxidation, which forces T cells into glycolysis while impairing fatty acid oxidation, causing mitochondrial damage and terminal exhaustion (32). This chronic nutrient deprivation becomes epigenetically fixed: metabolites such as lactate drive histone lactylation, remodeling the chromatin landscape of immune cells to sustain an exhausted transcriptional program (38).

#### 2.2.2 Neuroendocrine suppression: stress hormones as extrinsic checkpoints

Superimposed on this metabolic deprivation, the cachexia-associated neuroendocrine stress response imposes an additional layer of T cell suppression. Cancer cachexia is accompanied by chronic hyperactivation of the hypothalamic-pituitary-adrenal (HPA) axis and the sympathetic nervous system, releasing systemic stress hormones that act within the TIME as potent extrinsic immune checkpoints. Glucocorticoids induce a gradient of exhaustion in CD8+ T cells through the glucocorticoid receptor (GR/NR3C1). Single-cell transcriptomic analyses have revealed that GR cooperates with the non-canonical transcription factor RUNX3 to drive CD8+ T cell dysfunction, transactivating multiple checkpoint receptors including PD-1, LAG-3, and TIM-3 (39, 40). Additionally, GCs promote T cell apoptosis by upregulating the pro-apoptotic BH3-only protein Bim (39). GCs also impair antigen presentation through the ACBP/DBI–GABA-A receptor circuit, a pathway in which glucocorticoid-induced acyl-CoA binding protein (ACBP/DBI) acts on GABA-A receptors to suppress type I IFN signaling in dendritic cells, thereby limiting naive T cell priming (41).

Simultaneously, the surge in systemic catecholamines acts through β-adrenergic receptors to enforce immune tolerance. Terminally exhausted CD8+ T cells upregulate the β1-adrenergic receptor (ADRB1); upon catecholamine engagement, these cells exhibit marked suppression of cytokine production and proliferation (33). Concurrently, β2-adrenergic signaling blocks the metabolic reprogramming essential for T cell activation, driving a shift from a Th1 to a Th2 phenotype and compromising IFN-γ secretion (42).

#### 2.2.3 Metabolic asymmetry: Treg and MDSC adaptation

In contrast to effector T cells that depend on glucose, immunosuppressive Tregs and MDSCs exhibit substantial metabolic plasticity. They persist and expand in the cachectic TIME by upregulating lipid transporters and shifting their primary energy source to FAO and oxidative phosphorylation (OXPHOS).

Cachexia-driven lipolysis of systemic adipose tissue provides an abundant supply of FFAs to the TIME. Tregs selectively upregulate the fatty acid scavenger receptor CD36, a process catalyzed by the FOXP3/HAT1 epigenetic axis. CD36-mediated lipid uptake activates PPAR-β signaling, enhancing mitochondrial fitness and allowing Tregs to utilize the abundant local lactate as an alternative fuel source (43, 44).

Similarly, MDSCs undergo a metabolic switch toward FAO upon entering the TIME, driven by STAT3-dependent upregulation of lipid transporters such as FATP2. FATP2 facilitates the uptake of arachidonic acid, which is converted into prostaglandin E2 (PGE2) via COX-2, generating an immunosuppressive environment that further inhibits CD8+ T cell cytotoxicity and NK cell function (45, 46). Cachexia-associated stress hormones, acting through β2-adrenergic receptors, directly stimulate this glycolytic-to-OXPHOS shift in MDSCs, directly linking the systemic neuroendocrine stress of cachexia with local immunosuppressive metabolic adaptations (34).

### 2.3 Molecular crosstalk and emerging frontiers

The systemic deterioration observed in cancer cachexia is governed by a coordinated network of molecular signals. While classical inflammatory pathways initiate tissue catabolism, emerging evidence has identified an additional set of metabolic-immune factors and non-apoptotic cell death mechanisms that epigenetically and metabolically stabilize the immunosuppressive state.

#### 2.3.1 Classic inflammatory-catabolic hubs: the NF-κB and JAK2/STAT3 axes

The IL-6/JAK2/STAT3 and TNF-α/NF-κB signaling pathways function as dual-function molecular hubs in the cachectic setting. In skeletal muscle, IL-6 trans-signaling activates STAT3, which cooperates with NF-κB to transcriptionally upregulate the muscle-specific E3 ubiquitin ligases MuRF1 (TRIM63) and Atrogin-1 (FBXO32), directly executing myofibrillar protein degradation (47, 48). Concurrently, within the TIME, these same pathways act as central regulators of immune evasion. STAT3 drives the expansion of MDSCs through the IRF8-C/EBPβ axis and upregulates arginase-1 (Arg-1), while NF-κB directly binds the CD274 promoter to induce PD-L1 expression on both tumor cells and macrophages (49, 50). These two pathways engage in a bidirectional feed-forward loop: NF-κB transcribes IL-6, which further sustains STAT3 activation, sustaining a self-reinforcing cycle of continuous muscle wasting and T cell exhaustion.

#### 2.3.2 The GDF15/GFRAL central node: linking anorexia and immune tolerance

Beyond these classical cytokine pathways, tumor cells subjected to STAT3/NF-κB-mediated stress also secrete GDF15, a divergent member of the TGF-β superfamily, which functions as a distal amplifier of the cachexia-immune evasion circuit. Growth Differentiation Factor 15 (GDF15) has emerged as a key dual-function mediator linking neuroendocrine wasting with local immune suppression. Secreted abundantly by stressed tumor cells and tumor-associated macrophages (TAMs), GDF15 acts centrally by binding to the GFRAL/RET receptor complex in the hindbrain (area postrema). This central activation drives anorexia and stimulates peripheral sympathetic outflow, inducing adipose tissue lipolysis and accelerating muscle wasting independently of food intake (51, 52). Peripherally, GDF15 acts as an immune checkpoint: it impairs dendritic cell maturation, blocks LFA-1-mediated CD8+ T cell tumor infiltration, and interacts with CD48 on T cells to stabilize FOXP3, driving Treg expansion (53, 54). This integrated tumor-immune-neural circuit positions GDF15 as a core vulnerability, a concept clinically supported by the anti-cachexia efficacy of the GDF15-neutralizing antibody ponsegromab in recent phase 2 trials (55).

#### 2.3.3 Lactate signaling and epigenetic reprogramming via histone lactylation

Tumor-derived lactate functions as a multifunctional mediator in cachexia. Extracellularly, lactate binds to the GPR81 receptor on dendritic cells and tumor cells, suppressing MHC-II presentation while inducing PD-L1 and the Treg-recruiting chemokine CX3CL1 (56, 57). Intracellularly, lactate substantially alters the epigenetic landscape through histone lactylation (Kla), a recently identified post-translational modification. H3K18la deposition directly stimulates the transcription of immunosuppressive genes. In macrophages, lactylation serves as a metabolic clock driving M2 polarization (upregulating Arg1); in T cells, it stabilizes FOXP3 for Treg lineage commitment while mediating TOX-induced terminal exhaustion in CD8+ T cells (58, 59). Concurrently, the shuttling of large lactate loads to the liver for gluconeogenesis (the Cori cycle) creates a futile, energy-consuming cycle that accelerates host tissue wasting. This hepatic recycling of lactate not only depletes host energy but also sustains systemic oxidative stress. The resulting accumulation of lipid peroxides in peripheral tissues provides a direct biochemical substrate for ferroptosis, a recently identified mechanism of muscle destruction in cachexia.

#### 2.3.4 Emerging frontier: the ferroptosis-inflammation-immunosuppression cycle

A mechanistically significant advance in cachexia pathogenesis is the recognition of ferroptosis—an iron-dependent, lipid peroxidation-driven form of cell death—as a primary mechanism of muscle destruction. Cachectic skeletal muscle exhibits marked iron metabolism dysregulation, characterized by ferritin accumulation and hepcidin-mediated ferroportin degradation. Tissue-infiltrating neutrophils deliver iron directly to wasting muscle via lipocalin-2 (LCN2), while tumor-derived extracellular vesicles transfer miRNAs (e.g., miR-203a-3p) that systematically dismantle the GSH/GPX4 antioxidant axis, resulting in extensive lipid peroxidation and muscle ferroptosis (60, 61).

Ferroptotic muscle cells do not undergo immunologically silent cell death. They release an immunogenic secretome containing DAMPs including HMGB1, decorin (DCN), 8-OHG, and oxidized phospholipids (OxPLs). These ferroptosis-derived DAMPs act as established “find-me” signals that engage macrophage pattern recognition receptors (AGER, TLR4, and the cGAS-STING pathway). This engagement triggers the NLRP3 inflammasome and NF-κB pathways, leading to the release of IL-1β and IL-6, which drive enhanced M2 macrophage polarization and MDSC recruitment (62, 63). Paradoxically, MDSCs themselves undergo a form of sublethal ferroptosis within the TIME, releasing oxidized phosphatidylcholines (OxPCs) and PGE2 that directly impair T cell proliferation (64). Consequently, muscle ferroptosis functions not only as a terminal mechanism of myofiber loss but also as the upstream trigger of a self-amplifying “ferroptosis-inflammation-immunosuppression” feedback loop.

## 3 TCM monomers: precision intervention at the molecular interface

The multifactorial pathogenesis of cancer cachexia—driven by a self-amplifying loop of metabolic wasting and immune evasion—renders single-target synthetic therapies largely ineffective. In contrast, TCM-derived monomers act as multi-target modulators. By engaging multiple shared signaling nodes concurrently, these compounds execute a “one compound, multiple targets” pharmacology that closely aligns with the complex biology of the cachectic TIME (65). Rather than merely antagonizing a single cytokine, TCM monomers exert context-dependent, bidirectional regulation at the immunometabolic interface, simultaneously protecting host tissues from catabolism while rendering tumor cells vulnerable to immune clearance.

### 3.1 Curbing core cachexia pathways: from protein degradation to ferroptosis

The intervention of TCM monomers in cachexia is anchored in their ability to dismantle the core catabolic machinery across skeletal muscle and adipose tissue, extending from classical ubiquitin-proteasome system (UPS) regulation to the control of non-apoptotic cell death—ferroptosis (**Table 1**).

**Table 1:** Summary of TCM-derived monomers targeting the immunometabolic interface in cancer cachexia.

Monomer	Chemical class	Experimental model & disease context	Primary target(s)/pathways	Immunometabolic mechanisms & effects	Ref.
Curcumin	Polyphenol	*In vitro* (breast/colon cancer lines, myoblasts) & *In vivo* (C26/LLC mouse cachexia models)	NF-κB/UPP axis; STAT3 (Cys259); GPX4/SLC7A11 (Nrf2)	Inhibits MuRF1/Atrogin-1 to preserve muscle density; blocks tumor PD-L1 transcription via orthosteric covalent STAT3 binding; activates anti-ferroptotic defense in host tissue while inducing oxidative stress in tumors.	(78, 96–98)
Andrographolide	Labdane diterpenoid	*In vitro* (H-Ras transformed cells, cancer cells) & *In vivo* (C26 mouse cachexia & tumor models)	NF-κB p50 (Cys62); Keap1 (Cys151)/Nrf2; NLRP3 inflammasome	Forms covalent adduct with p50 to reduce cachexigenic cytokines (TNF-α, IL-6); liberates Nrf2 to shield muscle from ferroptosis; triggers mitophagy to prevent NLRP3 assembly and MDSC expansion.	(103–107)
EGCG	Polyphenol	*In vitro* (T cells, MIN6 β-cells, cancer lines) & *In vivo* (tumor-bearing/C26 mice)	STAT3 (SH2 domain); LKB1/AMPK pathway; Labile iron pool	Directly binds STAT3 SH2 domain to reactivate CD8+ T cells; promotes mitochondrial fitness in T cells to lift metabolic constraints; acts as an iron chelator to protect muscle against ferroptosis.	(74, 108–110)
Ursolic acid	Pentacyclic triterpene	*In vivo* (fasting/C26/LLC muscle atrophy mice) & *In vitro* (C2C12 myotubes)	SIRT1/Akt/mTOR; FoxO3a/E3 ligases	Directly activates SIRT1 to suppress STAT3/NF-κB inflammation; enhances Akt/mTOR to promote protein translation and sequester FoxO3a, markedly downregulating MuRF1/Atrogin-1.	(66, 67)
Baicalein/Baicalin	Flavonoid	*In vitro* (TAMs, C2C12) & *In vivo* (LLC/B16 tumor & cachexia models)	Akt/Fbx32/MuRF1; PI3Kγ/NF-κB; TRAF2/RelB	Sustains Akt phosphorylation in muscle to block atrophy, while blocking PI3K/Akt in tumor cells; inhibits PI3Kγ or degrades TRAF2 to repolarize TAMs from M2 to tumoricidal M1 phenotype.	(68, 69, 75, 76, 86, 87)
Salidroside	Phenylethanoid glycoside	*In vitro* (TNF-α-treated C2C12 myotubes) & *In vivo* (C26 mouse cachexia model)	mTOR-MyHC axis	Directly activates mTOR-MyHC signaling to rescue myofibrillar protein synthesis under TNF-α stress.	(70)
Carnosol	Diterpene	*In vitro* (adipocytes, myotubes) & *In vivo* (LLC tumor-bearing cachectic mice)	AMPK/HSL/UCP1; NF-κB/MuRF1	Addresses the browning paradox: inhibits AMPK/HSL in WAT to halt futile thermogenesis and lipolysis; concurrently inhibits NF-κB in muscle to downregulate MuRF1.	(71)
Berberine	Isoquinoline alkaloid	*In vitro* (cancer cell lines) & *In vivo* (MC38/CT26 tumor-bearing mice)	COP9 signalosome 5	Selectively binds CSN5 to block PD-L1 deubiquitination, forcing its proteasomal degradation to promote T cell cytotoxicity.	(82)
Ginsenoside Rg3	Triterpene saponin	*In vitro* (cancer cells) & *In vivo* (mouse lung/melanoma tumor models)	EGFR/GSK3β pathway	Inhibits EGFR signaling to accelerate GSK3β-mediated phosphorylation and proteasomal degradation of PD-L1.	(83)
Artemisinin/DHA	Sesquiterpene lactone	*In vitro* (MDSCs, Tregs) & *In vivo* (tumor-bearing mice)	PI3K/AKT, MAPK, mTOR; FoxP3 expression	Shifts MDSCs away from immunosuppressive signature (downregulates Arg-1, IL-10, TGF-β); suppresses Treg expansion to restore the CTL-to-Treg ratio.	(88, 89)
Oridonin	Diterpenoid	*In vitro* (macrophages) & *In vivo* (inflammatory/tumor mouse models)	NLRP3 NACHT domain (Cys279)	Acts as a selective covalent inhibitor of NLRP3; physically blocks NLRP3-NEK7 interaction and halts inflammasome assembly to cut off the autoinflammatory loop.	(92)
Celastrol	Triterpenoid	*In vitro* (macrophages) & *In vivo* (C26 mouse cachexia/systemic inflammation)	NLRP3 palmitoylation	Prevents NLRP3 palmitoylation and subsequent Golgi translocation, reducing systemic IL-1β and host tissue wasting.	(93)
Resveratrol	Stilbenoid	*In vitro* (C2C12, neurotoxicity & cancer cells) & *In vivo* (C26/rat cachexia models)	Keap1-Nrf2 axis; NCOA4 ferritinophagy; ASC oligomerization	Promotes Nrf2 nuclear translocation to upregulate SLC7A11 and GPX4; downregulates NCOA4-mediated ferritinophagy to safely sequester iron; disrupts mitochondrial spatial arrangement required for ASC specks.	(77, 79, 94, 95)

#### 3.1.1 Modulating the anabolic-catabolic nexus: PI3K/Akt/mTOR and FoxO3/MuRF1/Atrogin-1

In cachectic skeletal muscle, the balance between protein synthesis and degradation is severely disrupted. TCM monomers recalibrate this balance by targeting the Akt nexus, which acts as a central molecular switch. Akt phosphorylation simultaneously activates mTORC1 to promote protein translation and phosphorylates the transcription factor FoxO3a, sequestering it in the cytoplasm to halt the transcription of E3 ubiquitin ligases.

Ursolic acid, a pentacyclic triterpene, functions as a multi-target muscle protective agent. It preserves muscle mass across multiple cancer models by directly activating SIRT1, which subsequently suppresses STAT3/NF-κB inflammatory signaling while enhancing Akt/mTOR activity, leading to a substantial downregulation of MuRF1 and Atrogin-1 (66, 67). Similarly, baicalein (a predominant flavonoid from Scutellaria baicalensis) mitigates body weight loss and muscle wasting by specifically sustaining Akt phosphorylation to block Fbx32/MuRF1 expression. Baicalein exhibits tissue-specific bidirectionality: while it activates PI3K/Akt in muscle to promote anabolism, it inhibits the same pathway in tumor cells to suppress proliferation (68, 69). Furthermore, salidroside directly activates the mTOR-MyHC axis, rescuing myofibrillar protein synthesis even under TNF-α-induced stress (70).

Beyond skeletal muscle, TCM monomers also target the adipose tissue compartment, where pathological lipolysis and WAT browning represent parallel drivers of cachexia-associated energy dissipation.

#### 3.1.2 Regulating lipolysis and pathological browning: the AMPK-UCP1 axis

Adipose tissue wasting in cachexia is driven by hyperactive lipolysis and pathological WAT browning, characterized by UCP1 upregulation that forces mitochondrial respiration toward futile, energy-wasting thermogenesis. AMPK occupies a context-dependent role in this process: whereas its activation in muscle can be protective, its pathological hyperactivation in cachectic WAT drives hormone-sensitive lipase (HSL) and UCP1 expression, fueling energy-wasting thermogenesis. This creates a critical browning paradox, in which a pathway that is metabolically beneficial in obesity becomes detrimental in cachexia.

TCM-derived diterpenes, such as carnosol, address this paradox through tissue-specific pathway modulation. In cachectic adipose tissue, carnosol markedly inhibits AMPK and HSL phosphorylation, preventing UCP1-mediated thermogenesis and lipolysis. Concurrently, in skeletal muscle, it inhibits NF-κB and downregulates MuRF1, providing simultaneous dual-organ protection (71). In parallel, curcumin shields adipose tissue by blocking the upstream β-adrenergic-stimulated cAMP/PKA/CREB signaling pathway, directly suppressing the transcriptional drive for lipolytic enzymes without disrupting basal host metabolism (72).

This apparent paradox—AMPK activation being protective in muscle yet detrimental in adipose tissue—is reconciled by tissue-specific downstream effector wiring. In skeletal muscle, AMPK primarily phosphorylates acetyl-CoA carboxylase (ACC) to enhance fatty acid oxidation and restore ATP production under energy stress, which is metabolically beneficial. In contrast, in WAT during cachexia, pathological AMPK hyperactivation directly phosphorylates and activates HSL independently of the canonical PKA pathway, driving futile thermogenesis and lipolysis. Consequently, the directional recommendation for AMPK modulation is not contradictory but strictly tissue-context-dependent: agents that activate AMPK in muscle (e.g., EGCG via LKB1, or BYD via SIRT1) support mitochondrial fitness, whereas agents that inhibit AMPK hyperactivation in WAT (e.g., carnosol) prevent energy-wasting lipolysis. We explicitly reconcile this distinction here to avoid misinterpretation and to emphasize that AMPK is not a universal on/off target but a node whose modulation must be decoded by the cellular metabolic landscape.

#### 3.1.3 Emerging frontier: anti-ferroptotic protection of skeletal muscle

The recent identification of ferroptosis as a dominant driver of cachectic muscle atrophy has unveiled a novel therapeutic frontier for TCM monomers. Cachectic muscle is highly susceptible to iron overload (driven by hepcidin-mediated ferroportin degradation and LCN2 influx) and glutathione depletion, leading to extensive lipid peroxidation. TCM-derived polyphenols—such as curcumin, epigallocatechin gallate (EGCG), baicalein, and resveratrol—are structurally capable of halting this ferroptotic cascade through four convergent strategies:

Direct Iron Chelation: Polyphenols directly shrink the labile iron pool (LIP). The β-diketone moiety of curcumin and the gallol group of EGCG form stable coordination complexes with Fe^2+^/Fe^3+^, directly suppressing the Fenton reaction and preventing hydroxyl radical generation (73, 74).

Radical-Trapping Antioxidant (RTA) Activity and ALOX Inhibition: Beyond scavenging reactive oxygen species (ROS), these compounds operate as potent RTAs. Baicalein, identified through natural product library screening, exhibits anti-ferroptotic potency superior to classical inhibitors such as ferrostatin-1. It achieves this by directly binding and inhibiting arachidonate lipoxygenases (ALOX12/ALOX15), blocking the enzymatic generation of toxic lipid hydroperoxides at the membrane level (75, 76).

Nrf2/GPX4/xCT Transcriptional Activation: TCM monomers epigenetically restore the collapsed antioxidant defense. Resveratrol and curcumin disrupt the Keap1-Nrf2 complex, promoting Nrf2 nuclear translocation. This upregulates the transcription of SLC7A11 (xCT) and GPX4, enhancing cystine import and repairing the phospholipid hydroperoxide reducing capacity of muscle cells (77, 78).

Iron Metabolism Regulation: Resveratrol further protects iron homeostasis by downregulating NCOA4-mediated ferritinophagy, ensuring that iron remains safely sequestered within ferritin complexes rather than being liberated into the pro-ferroptotic cytosol (79).

The anti-ferroptotic intervention of TCM monomers demonstrates marked context-dependent bidirectionality. While these compounds activate Nrf2 and chelate iron to protect host skeletal muscle from ferroptosis, they concurrently trigger ferroptosis in tumor cells. For instance, Tanshinone IIA activates the Nrf2/GPX4 axis to protect normal tissues from oxidative injury, yet in cancer cells, it utilizes p53-mediated suppression of SLC7A11 to induce lethal ferroptotic stress (80, 81). This capacity to selectively shield the host metabolic machinery while inducing metabolic catastrophe in the tumor positions TCM monomers as candidates for disrupting the cachexia-immunosuppression loop.

### 3.2 Reprogramming the immune microenvironment

Beyond arresting tissue catabolism, TCM monomers function as effective immunomodulators that counteract the immunosuppressive features of the TIME. By targeting key regulatory nodes such as PD-L1, STAT3, NF-κB, and the NLRP3 inflammasome, these compounds restore the anti-tumor efficacy of the host immune system while simultaneously dampening the systemic chronic inflammation that drives cachexia.

#### 3.2.1 Releasing the immune brake: downregulating PD-L1 expression

Overcoming immune checkpoint resistance is a primary objective in modern oncology, and TCM monomers achieve this by targeting PD-L1 at both the transcriptional and post-translational levels. Berberine, a classical isoquinoline alkaloid, selectively binds to the deubiquitinase COP9 signalosome 5 (CSN5). By inhibiting CSN5, berberine prevents the removal of ubiquitin chains from PD-L1, forcing its proteasome-dependent degradation and increasing tumor cell susceptibility to T cell cytotoxicity (82). Similarly, ginsenoside Rg3 targets PD-L1 at the post-translational level by inhibiting EGFR signaling, which accelerates GSK3β-mediated phosphorylation and subsequent proteasomal degradation of PD-L1 (83). At the transcriptional level, flavonoids such as isoliquiritigenin and luteolin markedly repress PD-L1 expression by inhibiting the JAK2/STAT3 signaling cascade, providing a molecular rationale for combining these monomers with clinical immune checkpoint blockade therapies (84, 85).

In addition to modulating checkpoint ligand expression on tumor cells, TCM monomers directly reprogram the immunosuppressive myeloid compartment, reversing the pro-tumor polarization of TAMs and the expansion of MDSCs.

#### 3.2.2 Reversing myeloid polarization: targeting the STAT3/NF-κB network

TAMs and MDSCs dictate the immunosuppressive tone of the TIME, relying on hyperactive STAT3 and NF-κB signaling. TCM monomers act as effective repolarizing agents that reverse these phenotypes.

Baicalin induces the repolarization of TAMs from the tumor-promoting M2 state to the tumoricidal M1 state by promoting autophagy-mediated degradation of TRAF2. This degradation activates the non-canonical NF-κB RelB/p52 pathway, resulting in increased production of pro-inflammatory cytokines (TNF-α, IL-12) and sustained tumor growth inhibition in macrophage-dependent models (86). Its aglycone form, baicalein, directly binds and inhibits PI3Kγ in macrophages, de-repressing the canonical NF-κB cascade to promote M1 polarization (87).

Artemisinin and its derivative dihydroartemisinin (DHA) have been characterized as effective MDSC modulators. By downregulating the PI3K/AKT and MAPK pathways while activating the mTOR signaling axis, artemisinin shifts MDSCs away from their immunosuppressive signature (downregulating Arginase-1, IL-10, and TGF-β) and markedly suppresses the expansion of Tregs by inhibiting FoxP3 expression. This rebalances the CD8+ CTL-to-Treg ratio, synergizing with anti-PD-L1 therapies in preclinical models (88, 89). This reprogramming of the myeloid compartment not only relieves local immune suppression but also reduces the systemic production of IL-6 and TNF-α, thereby partially uncoupling the TIME from the peripheral catabolic processes that sustain cachexia.

#### 3.2.3 Inhibiting the NLRP3 inflammasome: breaking the inflammation-cachexia circuit

The NLRP3 inflammasome represents a critical pathological bridge between tumor immunosuppression and cancer cachexia. In the TIME, tumor-derived IL-1β drives MDSC expansion and sustains an autoinflammatory IL-1β/IL-6/STAT3 loop (90). Simultaneously, in skeletal muscle, NLRP3 activation triggers mitochondrial ROS production and upregulates Atrogin-1, directly accelerating muscle wasting (91).

TCM monomers offer precise, multi-level intervention against this inflammasome. Oridonin, a diterpenoid, acts as a selective covalent inhibitor of NLRP3 by directly binding to cysteine 279 in its NACHT domain, physically blocking the NLRP3-NEK7 interaction and halting inflammasome assembly (92). Celastrol achieves similar suppression by preventing NLRP3 palmitoylation and subsequent Golgi translocation, reducing systemic IL-1β release (93). Curcumin and resveratrol disrupt the spatial arrangement of mitochondria required for ASC oligomerization and speck formation (94, 95). By inhibiting the NLRP3 inflammasome, these compounds limit the release of IL-1β and IL-18, reducing the inflammatory mediators that sustain the immunosuppressive TIME while simultaneously halting the cytokine-driven catabolism of host skeletal muscle.

### 3.3 Representative pharmacological modulators: bidirectional intervention at the immunometabolic interface

To illustrate the translational potential of the network perturber paradigm, several well-characterized TCM monomers exemplify the capacity for bidirectional intervention at the immunometabolic interface. These compounds concurrently suppress muscle catabolism, reprogram the immunosuppressive TIME, and protect host tissues against ferroptosis (**Figure 2**).

**Figure 2:**
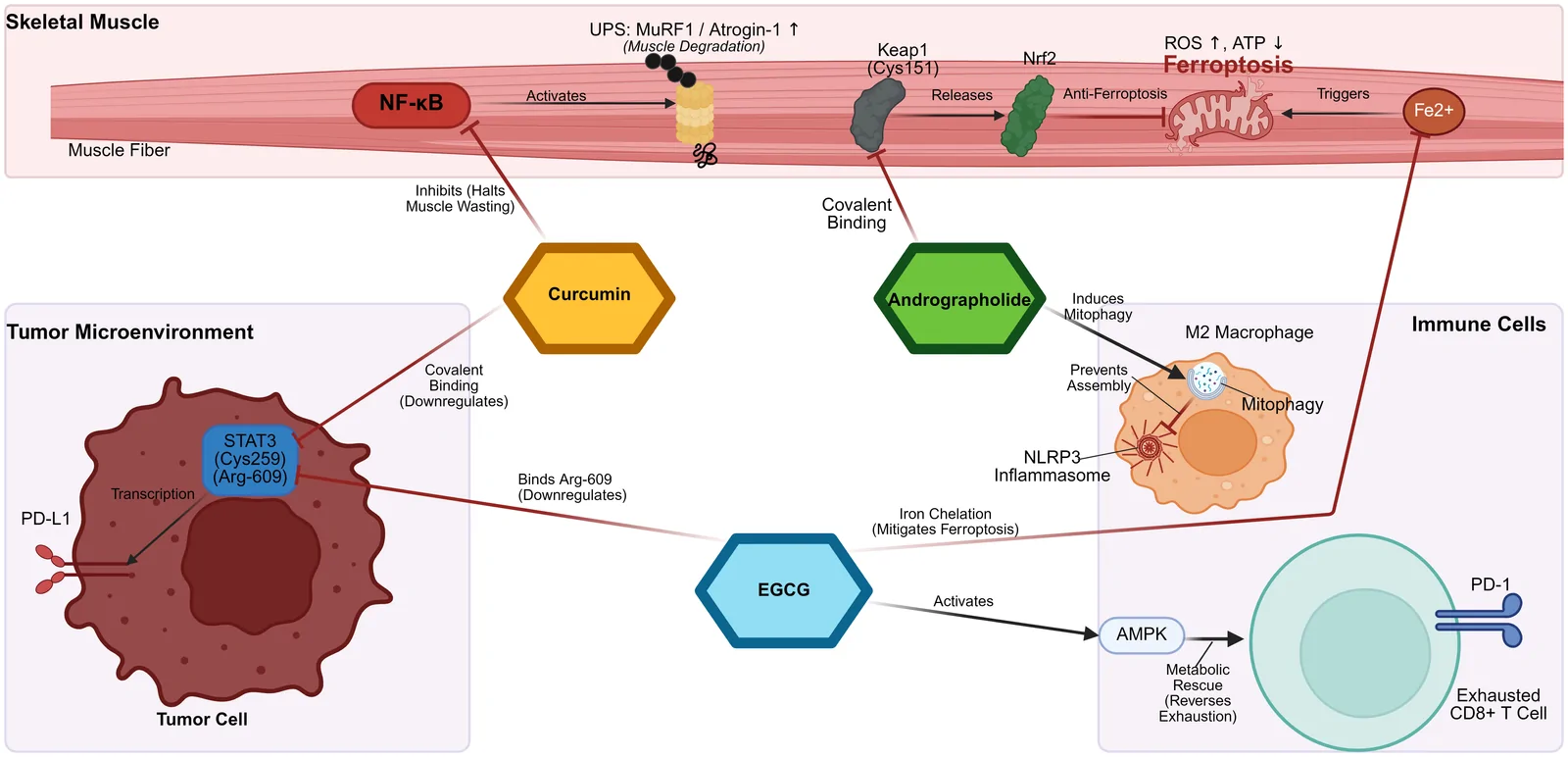
Multi-target pharmacological mechanisms of TCM-derived monomers in reversing cancer cachexia and immune evasion (Created with BioRender.com. Publication license agreement number: YV2A2ZRPTT. Citation: Jia Wang (2026). BioRender.com/a1yb3gk). Three representative TCM-derived monomers exert systemic immunometabolic effects via distinct targets. Curcumin ameliorates muscle wasting by inhibiting the NF-κB/proteasome pathway and reverses immune evasion by covalently binding STAT3 (Cys259) to downregulate tumor PD-L1 transcription. Andrographolide mitigates muscle ferroptosis by binding Keap1 (Cys151) to release Nrf2, while concurrently inducing mitophagy to prevent NLRP3 inflammasome assembly in macrophages. EGCG rescues exhausted CD8+ T cells via AMPK activation, limits muscle ferroptosis through iron chelation, and binds STAT3 to synergistically suppress PD-L1 expression. It should be noted that the parallel depiction of host-protective and tumor-toxic effects in this schematic is conceptual; the simultaneous occurrence of these opposite outcomes at identical *in vivo* exposures remains a hypothesis requiring dose-resolved validation (see Section 3.3).

#### 3.3.1 Curcumin: the pleiotropic hub modulator

Curcumin is among the most extensively studied dual-action agents in cancer cachexia. In the skeletal muscle compartment, curcumin directly inhibits the NF-κB/UPP axis. By preventing NF-κB p65 phosphorylation, it markedly suppresses the transcription of MuRF1 and Atrogin-1, preserving myofibrillar density and grip strength (96). Concurrently, within the TIME, preclinical studies in non-cachexia models indicate that curcumin may exert immunological effects through covalent interaction with cysteine 259 of STAT3 via its α,β-unsaturated carbonyl moiety (97). If applicable *in vivo*, this orthosteric blockade suppresses STAT3 phosphorylation, inhibiting M2 macrophage polarization and triggering the post-translational degradation of PD-L1 through CSN5 inhibition (98). Furthermore, curcumin acts as a RTA and disrupts the Keap1-Nrf2 complex, activating the GPX4/SLC7A11 anti-ferroptotic pathway in host tissues, while paradoxically exacerbating oxidative stress in tumor cells to induce ferroptotic cell death (78).

#### 3.3.2 Astragaloside IV: the gut-muscle-immune axis integrator

AS-IV, the principal cycloartane-type triterpenoid saponin from Astragalus membranaceus, provides systemic recalibration extending from the gut microbiome to the TIME. In cachectic muscle, AS-IV directly rescues the suppressed PI3K/Akt/mTOR anabolic cascade, simultaneously upregulating protein translation while downregulating TGF-β1/Smad-induced proteolysis (99). AS-IV also exerts indirect immunomodulatory effects by reshaping the gut microbiota, enriching *Alistipes* and *Prevotella* and increasing the production of short-chain fatty acids (SCFAs), particularly butyrate (100). Microbial butyrate has been independently shown to act as an epigenetic modifier that promotes a FOXO1-driven stemness program in CD8+ T cells, enhancing IFN-γ production and anti-tumor cytotoxic capacity (101). Concurrently, AS-IV maintains systemic iron homeostasis and mitigates lipid peroxidation by suppressing TFR1 and upregulating FTH1 and GPX4, thereby protecting host tissues from cachexia-induced ferroptosis (102).

#### 3.3.3 Andrographolide: covalent precision pharmacology

Andrographolide, a labdane diterpenoid, exhibits covalent precision pharmacology. Its anti-catabolic efficacy in muscle is mediated by its ability to form a covalent adduct with cysteine 62 (Cys62) of the NF-κB p50 subunit, blocking its DNA-binding activity and reducing the systemic levels of cachexigenic cytokines, including TNF-α and IL-6 (103). In the immune compartment, andrographolide inhibits the NLRP3 inflammasome through multiple mechanisms: it triggers PIK3CA/AKT-mediated mitophagy to clear damaged mitochondria and activates the miR-155/Nrf2/TXNIP axis, thereby reducing the IL-1β required for MDSC expansion (104, 105). Furthermore, andrographolide acts as a Michael acceptor that covalently modifies Cys151 on Keap1. This modification liberates Nrf2 to activate an antioxidant and anti-ferroptotic transcriptional program, including HO-1 and TRX2, thereby protecting normal muscle tissue while inducing p53-mediated ferroptotic cell death in colorectal and lung cancer cells (106, 107).

#### 3.3.4 Epigallocatechin gallate: epigenetic and metabolic reprogramming

EGCG, the major polyphenol in green tea, counteracts the cachexia-immunosuppression loop through targeted protein interactions and metabolic reprogramming. Immunologically, structural modeling and *in vitro* studies suggest that EGCG can bind the SH2 domain of STAT3 to suppress the JAK/STAT1/IRF1 signaling cascade. This inhibits PD-L1 and PD-L2 transcription, reactivating CD8+ T cells with an efficacy comparable to that of anti-PD-1 monoclonal antibodies in preclinical models (108, 109); nevertheless, these specific biochemical observations warrant cautious extrapolation to complex cachectic host tissues. Metabolically, EGCG suppresses aerobic glycolysis in tumor cells through dual PI3K/mTOR inhibition, while in CD8+ T cells it promotes mitochondrial fitness via LKB1/AMPK activation, mitigating the metabolic constraints imposed by the TIME (110). Finally, owing to its catechol and gallol moieties, EGCG functions as an effective iron chelator, neutralizing the LIP, intercepting lipid peroxyl radicals, and activating the Nrf2/HO-1 axis, thereby conferring protection against ferroptosis in normal host tissues (74).

#### 3.3.5 Reconciling context-dependent signaling and the tissue-selectivity hypothesis

A critical nuance in monomer pharmacology lies in the context-dependent and dose-dependent nature of signaling pathways. The hypothesis that a single monomer simultaneously protects host tissues (via antioxidant pathways) while selectively inducing oxidative stress in tumor cells requires careful dose–response consideration. In most preclinical systems, the concentrations required for direct covalent binding to transcription factors such as STAT3 or Keap1 (typically in the low-micromolar range *in vitro*) are substantially higher than those needed for systemic suppression of NF-κB or NLRP3 inflammasome activity. Consequently, at physiologically achievable plasma levels following standard oral administration, the “tumor-toxic” ferroptotic effect may not occur concurrently with the “host-protective” anti-ferroptotic effect. This does not invalidate the network-modulator concept but reframes it as a context- and dose-dependent continuum rather than a binary switch. Direct proof of simultaneous opposite redox outcomes within the same organism at identical exposures remains an open question requiring side-by-side, dose-resolved pharmacokinetic–pharmacodynamic studies.

### 3.4 Pharmacokinetic bottlenecks and translational considerations

Despite the compelling mechanistic bioactivities reported for TCM monomers, a major hurdle in their clinical translation is their unfavorable pharmacokinetic (PK) profile. Monomers such as curcumin, EGCG, and andrographolide frequently exhibit inherently poor aqueous solubility, rapid gastrointestinal degradation, and extensive first-pass hepatic metabolism. Consequently, while cell culture models frequently employ micromolar concentrations (e.g., 10–50 μM) to demonstrate direct target binding and pathway inhibition, standard oral administration in humans yields systemic plasma concentrations that fall far below these threshold doses. Therefore, *in vivo* mechanistic assertions derived from high-dose preclinical setups must be interpreted with extreme caution. To overcome these systemic exposure limits and bridge the gap between preclinical promise and patient benefit, future translational research must integrate advanced delivery systems—such as lipid nanoparticles or phospholipid complexes—designed to enhance oral bioavailability and tissue-targeted accumulation.

## 4 TCM formulas: systemic recalibration via the gut-muscle-immune axis

While TCM monomers provide targeted intervention against specific molecular nodes, classical multi-herb formulas offer a distinct therapeutic paradigm: systemic recalibration. Cancer cachexia and its associated immunosuppression are fundamentally network-level diseases driven by the collapse of inter-organ communication. TCM formulas, guided by the principles of “fortifying the spleen” and “fu zheng qu xie” (reinforcing healthy qi to eliminate pathogenic factors), function as network-level modulators. Emerging pharmacological evidence demonstrates that these multi-component formulations produce coordinated regulation across the gut-muscle-immune axis, simultaneously remodeling the gut microbiome, suppressing muscle catabolism, and restoring immune function within the TIME in ways that single-target synthetic agents have not replicated (**Table 2**) (12, 111). This gut-centric perspective is further supported by a recent comprehensive review positioning the gut microbiota as a master regulator of the neuro-immune-metabolic crosstalk in cachexia (112), providing a broader contextual framework for the formula-based interventions discussed below.

**Table 2:** Summary of chemically characterized TCM formulas providing systemic recalibration via the gut-muscle-immune axis.

TCM formula	Key botanical components	Validation method & quality markers	Experimental model & disease context	Systemic targets & multi-organ mechanisms	Final therapeutic outcome	Ref.
Sijunzi Decoction (SJZD)	Radix Ginseng, Rhizoma Atractylodis Macrocephalae, Poria, Radix Glycyrrhizae	HPLC or LC-MS/MS; Validated via non-digestible polysaccharide fractions.	*In vivo* (C26/CT26 mouse cachexia & tumor models) & *In vitro* (TAMs, gut microbiota cultures)	Gut: Enriches butyrate-producing commensals (*Butyricimonas*, *Akkermansia*) to boost SCFAs and restore ZO-1/occludin barrier. TIME: Reduces intratumoral lactate and downregulates H3K18 lactylation in TAMs to suppress M2 polarization. Muscle: Downregulates Atrogin-1 and MuRF1 to rescue myatrophy.	Restores gut-muscle-immune axis homeostasis; suppresses muscle wasting and reactivates CD8+ T cells.	(119–121)
Buzhong Yiqi Decoction (BYD)	Radix Astragali, Radix Glycyrrhizae, Radix Angelicae Sinensis, etc.	UHPLC-Q-Orbitrap HRMS/LC-MS/MS fingerprinting.	*In vivo* (Colon-26/LLC mouse cachexia models) & Clinical observational cohort	Mitochondria: Activates the AMPK/SIRT1/PGC-1α signaling axis. Muscle: Elevates intracellular ATP levels while concurrently inhibiting JNK/c-JUN-mediated muscle proteolysis. TIME: Suppresses TNF-α, IL-6, and NF-κB cascades.	Reverses energy failure and cancer-related fatigue; breaks redundant inflammatory-catabolic networks.	(122, 123, 127)
Danggui Buxue Tang (DBT)	Radix Astragali, Radix Angelicae Sinensis	HPLC or LC-MS/MS fingerprinting.	*In vivo* (Anemic tumor-bearing mice) & *In vitro* (Hematopoietic progenitor cells)	Hematopoiesis: Activates HIF-1α/Raf/MEK/ERK pathway in hematopoietic tissues to stimulate erythropoiesis. TIME: Suppresses pathological HIF-1α/VEGF-driven angiogenesis and hypoxic immunosuppression.	Corrects tumor-related anemia, restores tissue oxygen delivery, and alleviates hypoxic barriers in the TIME.	(124, 125)
Wumei Pills/Modified Pulsatilla Decoction	Fructus Mume, Radix Pulsatillae, etc.	Chromatographic fingerprinting; LC-MS/MS validated active phytoconstituents.	*In vivo* (5-FU/Irinotecan/CPT-11 induced chemotherapy mucositis & cachexia mouse models)	Gut Barrier: Restores ZO-1 and claudin-1 expression in intestinal epithelium damaged by aggressive therapies (e.g., 5-FU). Systemic Circulation: Prevents luminal endotoxin (LPS) translocation into circulation. Muscle: Blunts TLR4/NF-κB signaling in myocytes.	Severs the chemotherapy/radiotherapy-induced mucositis-endotoxemia-muscle degradation vicious cycle.	(147)

For these systemic effects to be evaluated with scientific rigor, a critical barrier must first be addressed: the chemical characterization of the formulas under investigation.

### 4.1 Prerequisites for inclusion: chemical fingerprinting and quality control

A persistent limitation in TCM formula research has been the reliance on chemically undefined decoctions, which compromises the reproducibility of pharmacological evaluation. We advocate that rigorous chemical characterization remain a critical desideratum for evaluating multi-herb formulas in translational oncology. While contemporary quality control frameworks—such as the Consensus Statement on the Phytochemical Characterisation of Medicinal Plant Extracts (113)—emphasize the necessity of chromatographic fingerprinting (e.g., via HPLC or LC-MS/MS) to ensure batch-to-batch reproducibility, we acknowledge that for several of the cited preclinical studies on specific formulas (e.g., SJZD and BYD), detailed chromatographic fingerprinting of the specific experimental batches was not explicitly reported in the original publications. Therefore, this section serves primarily as a quality control framework for future translational studies, rather than a retrospective validation of all cited literature. In the present review, multi-herb formulas are evaluated primarily on the strength of their demonstrated systemic biological activity, while highlighting quality markers (Q-markers) as an essential prerequisite for future standardized clinical translation.

Such analytical approaches—employing techniques such as UHPLC-Q-Orbitrap HRMS combined with network pharmacology (114)—enable batch-to-batch consistency and permit the identification of specific quality-markers (Q-markers). By meeting these regulatory-grade frameworks, as delineated by the U.S. FDA botanical drug guidance (115) and the European Medicines Agency (EMA) specifications for herbal medicinal products (116), the systemic effects discussed in the following sections can be attributed to reproducible, quantifiable phytochemical matrices rather than undefined biological variables.

#### 4.1.1 Distinguishing in silico predictions from *in vivo* validations

A critical methodological distinction must be made between network pharmacology predictions and direct experimental proof. Many foundational rationales for multi-herb formulas rely heavily on in silico target prediction, which frequently highlights non-specific, high-connectivity hub targets such as AKT1, IL-6, TNF, and TP53. While these bioinformatic analyses map potential therapeutic networks, they do not constitute definitive proof of physical target engagement. Throughout the following sections and as summarized in **Table 2**, we explicitly distinguish between predicted multi-target networks and those pathways directly validated *in vivo*—such as the Western blot-confirmed suppression of muscle MuRF1/Atrogin-1, 16S rRNA-sequenced restoration of SCFA-producing gut microbiota, and flow cytometry-quantified CD8+ T cell reinvigoration in cachectic tumor models.

### 4.2 Core mechanistic axis: the gut-muscle-immune axis

In the advanced stages of cancer cachexia, the collapse of inter-organ communication triggers a systemic physiological crisis. The pathology is rooted in the gut-muscle-immune axis. Dysbiosis of the gut microbiota—characterized by depletion of SCFA-producing commensals and an overgrowth of pathobionts—directly compromises intestinal tight junctions (ZO-1, occludin). This barrier breach leads to endotoxemia, permitting lipopolysaccharides (LPS) to translocate into systemic circulation. The resulting activation of the TLR4/NF-κB cascade produces a dual effect: it triggers extensive MuRF1-mediated skeletal muscle proteolysis while simultaneously inducing a state of marked immune suppression characterized by T cell exhaustion (see again **Figure 1**) (117, 118).

Interception of this cascade at the level of gut barrier integrity and microbial metabolite production represents a mechanistic entry point for classical spleen-fortifying formulas. These formulas, validated by LC-MS/MS fingerprinting, function as systemic calibrators. Among them, Sijunzi Decoction (SJZD) exemplifies the systemic recalibration of the gut-muscle-immune axis.

#### 4.2.1 SJZD: microbiota-metabolite-immune calibration

The non-digestible polysaccharide fractions of SJZD bypass the upper gastrointestinal tract to act as selective prebiotics in the colon. These macromolecules enrich butyrate-producing commensals (e.g., *Butyricimonas*, *Akkermansia*), increasing SCFA production (119, 120). This SCFA restoration repairs the intestinal barrier by upregulating ZO-1 and occludin, thereby reducing the systemic influx of endotoxins and attenuating the subsequent TLR4/NF-κB-driven inflammatory response. A recent study demonstrated that SJZD mitigates cachexia-associated muscle atrophy through epigenetic-metabolic reprogramming: it reduces intratumoral lactate accumulation, thereby downregulating histone H3K18 lactylation (H3K18la) in TAMs. This suppresses M2 macrophage polarization, downregulates the muscle E3 ligases Atrogin-1 and MuRF1, and restores CD8+ T cell function (121).

#### 4.2.2 Restoring energy homeostasis and oxygen delivery: Buzhong Yiqi Decoction and Danggui Buxue Tang

Energy failure and tissue hypoxia are cardinal hallmarks of cachexia. Buzhong Yiqi Decoction (BYD; known as Hochuekkito in Japan and Bojungikki-tang in Korea) addresses this energy deficit by activating the AMPK/SIRT1/PGC-1α signaling axis. This activation promotes mitochondrial biogenesis and elevates intracellular ATP levels, attenuating cancer-related fatigue while inhibiting JNK/c-JUN-mediated muscle proteolysis (122, 123). Danggui Buxue Tang (DBT) modulates the hypoxic response: it corrects tumor-related anemia by activating the HIF-1α/Raf/MEK/ERK pathway in hematopoietic tissues to stimulate erythropoiesis, while suppressing pathological HIF-1α/VEGF-driven angiogenesis and immunosuppression within the hypoxic TIME (124, 125).

#### 4.2.3 Integrative effect: multi-target network modulation

The pharmacological basis of these multi-herb formulas resides in their capacity as multi-target network modulators. Rather than producing complete blockade of a single node, which can activate compensatory resistance pathways, TCM formulas contain multiple bioactive phytochemicals that exert simultaneous, partial inhibition across interconnected cachexigenic networks (126). Network pharmacology analyses indicate that formulas such as BYD concurrently suppress TNF-α, IL-6, and NF-κB while reprogramming metabolic pathways in the microenvironment (127). This multi-component, multi-target mode of action may offer advantages against the redundant signaling networks that characterize cancer cachexia, compared with monotherapy approaches.

## 5 Clinical perspectives: TCM as an adjuvant in the immunotherapy era

Preclinical mechanistic data have established TCM as an immunometabolic modulator. Its translational value, however, depends on clinical evidence. ICIs have transformed oncology, yet primary resistance and immune-related adverse events (irAEs) limit their efficacy. Notably, cachexia itself is an independent predictor of poor ICI response, as systemic inflammation and nutritional depletion compromise both T cell fitness and drug tolerability (4). This association is further substantiated by a systematic review demonstrating that pre-treatment weight loss and sarcopenia are consistent predictors of poorer survival and response to ICIs (128). Theoretically, TCM interventions are positioned to act as systemic adjuvants that simultaneously address host nutritional/functional deficits and reprogram the immunosuppressive TIME, thereby potentiating ICI efficacy through the synergistic sensitization mechanism illustrated in **Figure 3**.

**Figure 3:**
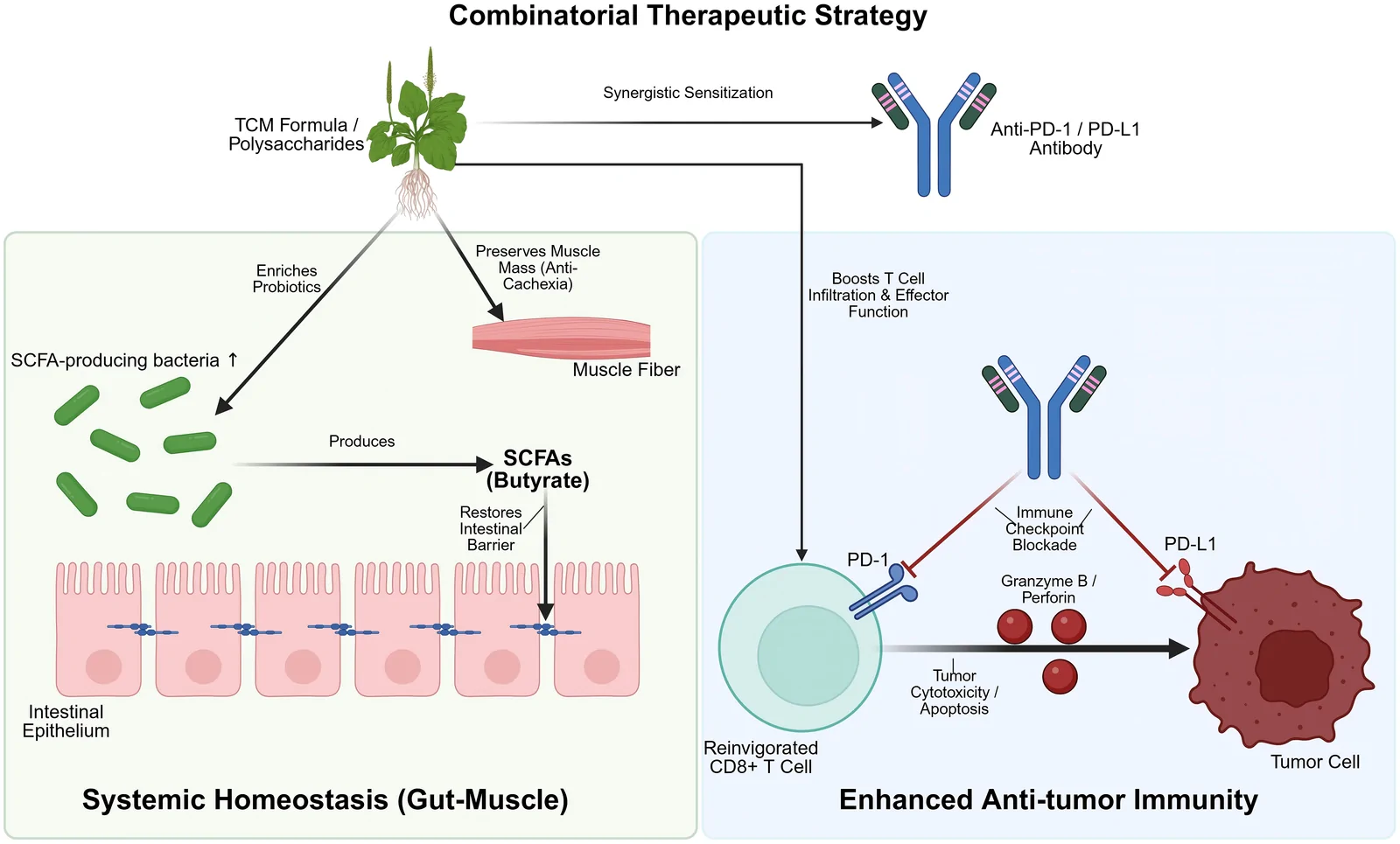
Synergistic combinatorial strategy of TCM formulas and ICIs (created with BioRender.com. Publication license agreement number: EO2A2ZS3NR. Citation: Ni, B (2026). BioRender.com/cmk306w). Holistic intervention with TCM formulas (e.g., polysaccharides) restores systemic homeostasis by enriching SCFA-producing bacteria to repair the intestinal epithelial barrier and preserving skeletal muscle mass against cachexia. Concurrently, TCM boosts T cell infiltration and effector functions. Through “synergistic sensitization,” combining TCM with ICIs (anti-PD-1/PD-L1 antibodies) for immune checkpoint blockade effectively reinvigorates exhausted CD8+ T cells. These reinvigorated T cells secrete granzyme B and perforin, ultimately inducing potent tumor cytotoxicity and apoptosis.

Recent meta-analyses have attempted to pool the efficacy of TCM combined with ICIs. However, a rigorous appraisal must first emphasize a critical caveat: the underlying primary literature synthesized in these meta-analyses is heavily dominated by small-scale, single-center, open-label, and retrospective studies. Such designs carry substantial risks of severe confounding variables, reporting bias, and healthy-user bias—as patients capable of adhering to continuous oral herbal regimens often possess superior baseline physical reserves. With this methodological caveat explicitly in mind, pooled estimates suggest that the integration of TCM with ICIs may produce clinically meaningful benefits: the combination improves the Objective Response Rate (ORR) (RR 1.34) and extends OS by approximately 1.5 months compared with ICIs alone, accompanied by an 18% relative risk reduction (RRR) in adverse events (RR 0.82, 95% CI 0.69–0.97) (129). These effect sizes, while encouraging, must be interpreted with extreme caution, and they underscore the urgent need for high-quality randomized controlled trials.

### 5.1 Enhancing efficacy: restoring eligibility and optimizing response

The clinical utility of TCM as an ICI adjuvant operates across two temporal windows: pre-treatment eligibility restoration and on-treatment immune optimization.

#### 5.1.1 Restoring immunotherapy eligibility by reversing cachexia

A clinically relevant consideration in immuno-oncology is that patients with an ECOG performance status (PS) ≥2, marked sarcopenia, or hypoalbuminemia—classic manifestations of cachexia—are frequently not candidates for ICI therapy due to historically poor survival outcomes and heightened toxicity risks (130, 131). Pretreatment nutritional status is, however, increasingly recognized as a modifiable variable (132).

TCM formulas based on “fortify the spleen and replenish Qi” principles target this variable. Multiple meta-analyses indicate that the addition of TCM to standard cancer care improves KPS/ECOG scores by a mean difference of 6 to 13 points, sufficient to shift patients from the PS 2–3 range to the PS 0–1 range (133, 134). In clinical practice, interventions such as modified Sijunzi Decoction combined with enteral nutrition have been shown to improve hypoalbuminemia and lower the CRP/albumin ratio (135). By addressing the inflammatory-nutritional deficit and preserving lean body mass (LBM), TCM-based prehabilitation may enable patients with cachexia to regain ICI eligibility, providing a window for otherwise excluded patients to receive immunotherapy.

#### 5.1.2 Optimizing clinical response: modulating deep immunosuppression

Once a patient is receiving ICIs, TCM adjuvants may optimize the clinical response by modulating the immunosuppressive networks that limit the efficacy of PD-1/PD-L1 blockade alone. Clinical and translational evidence increasingly supports an association between TCM administration and the reduction of peripheral and intratumoral inhibitory cell populations.

In a randomized pilot trial of patients with advanced non-small cell lung cancer (NSCLC) receiving atezolizumab, administration of Bojungikki-tang (BJIKT) was associated with a decrease in PD-1^+^CD8+ T cells, a marker of T cell exhaustion, and an increase in functional NK cells, correlating with numerically superior disease control rates (136). In breast cancer, clinical cohort analyses have shown that the Shugan Jianpi Formula reduces the peripheral abundance of MDSCs and limits MDSC-induced T cell apoptosis through JAK/STAT signaling suppression (137). Complementing these cellular effects, specific TCM monomers, including curcumin and baicalein, exhibit direct enzymatic inhibition of indoleamine 2,3-dioxygenase 1 (IDO1). By blocking the tryptophan-kynurenine pathway, these compounds reduce kynurenine-driven AhR activation and limit the availability of signals required for Treg differentiation (138, 139). Through these mechanisms—suppressing MDSCs, downregulating IDO activity, and reducing Treg proportions—TCM adjuvants modulate the TIME immunosuppressive milieu, potentially enhancing tumor sensitivity to immune checkpoint blockade.

While these immunomodulatory effects may enhance anti-tumor efficacy, TCM formulations also have the potential to mitigate the toxicity of ICIs and conventional therapies, as discussed in the following section.

### 5.2 Mitigating toxicity: alleviating treatment-related adverse effects

While enhancing therapeutic efficacy is important, the parallel objective of adjuvant TCM is to mitigate the treatment-related toxicities of modern oncology regimens. TCM formulas have the capacity to protect host tissues from treatment-related injury without antagonizing the core antineoplastic mechanisms.

#### 5.2.1 Managing irAEs: the myocarditis/myositis conundrum

The use of ICIs has been associated with a spectrum of irAEs. Among these, ICI-induced myositis and myocarditis are associated with high mortality, often presenting as overlapping syndromes driven by autoreactive CD8+ T cells and macrophage infiltration into striated muscle (140). A clinical challenge in advanced cancer is differentiating acute ICI-induced myositis from the onset of cancer cachexia-induced muscle wasting. While cachexia manifests as a gradual loss of lean mass with normal creatine kinase (CK) levels, ICI-myositis typically presents acutely within the first 30 days of treatment, characterized by elevated CK, active myopathic EMG changes, and muscle edema on MRI (141, 142). This distinction is clinically consequential, as it determines whether the patient requires immunosuppressive intervention or nutritional-catabolic support.

TCM formulas and natural compounds have shown potential to address both scenarios through context-dependent immunomodulation. Preclinical models demonstrate that compounds such as sinomenine and berberine target the TLR4/MyD88/NF-κB/NLRP3 inflammasome axis—signaling pathways implicated in ICI-driven cardiomyocyte apoptosis and inflammatory myofiber necrosis (143, 144). By shifting the local cytokine milieu (upregulating IL-10 and reducing pathological IL-6 and CXCL9), TCM adjuvants have the potential to attenuate the localized inflammation of irAEs without broadly suppressing the systemic T cell responses required for tumor control.

#### 5.2.2 Alleviating chemotherapy/radiotherapy-induced mucositis: severing the vicious cycle

Chemotherapy and pelvic/abdominal radiotherapy damage the intestinal epithelium, downregulating tight junction proteins (ZO-1, occludin) and depleting mucin-2. This chemotherapy-induced mucositis triggers translocation of bacterial endotoxins into the systemic circulation (145). Translocated LPS binds to TLR4 on skeletal myocytes, initiating TLR4/NF-κB signaling that upregulates Atrogin-1 and MuRF1, directly causing muscle protein degradation (146). Thus, treatment-induced mucositis contributes to the progression of cancer cachexia.

Classical TCM formulas, validated by modern chromatographic fingerprinting, interrupt this pathological sequence at the mucosal barrier. Wumei Pills and Modified Pulsatilla Decoction—whose active phytoconstituents have been characterized via LC-MS/MS—restore ZO-1 and claudin-1 expression in 5-FU-treated intestinal epithelium, restoring intestinal barrier integrity and preventing LPS translocation (147). Furthermore, purified formula-derived monomers such as 6-shogaol (a bioactive constituent of Banxia Xiexin Tang) have been shown to reduce circulating D-lactic acid and endotoxin levels following irinotecan exposure, while rebalancing the Th17/Treg ratio in the lamina propria (148). By protecting the mucosal barrier and reducing the subsequent LPS/TNF-α/IL-6-driven inflammatory response, these chemically characterized TCM interventions sever the mechanistic link between gastrointestinal toxicity and systemic cachexia, preserving the host metabolic integrity during aggressive oncological treatment.

### 5.3 Safety liabilities, quality concerns, and herb-drug interactions

A balanced evaluation of TCM formulas and monomers in clinical oncology requires rigorous consideration of their safety profiles and potential drug interactions. Despite their multi-target therapeutic promise, several critical liabilities must be recognized:

Toxicity and Quality Control: The administration of complex herbal extracts carries inherent risks of herb-induced liver injury (DILI) and nephrotoxicity, particularly in cachectic patients with compromised metabolic organ clearance. Furthermore, batch-to-batch variation, contamination with heavy metals, pesticides, or accidental adulteration represent persistent quality control challenges that necessitate strict adherence to Good Agricultural and Collection Practices.

Pharmacokinetic HDIs: Many active phytoconstituents (e.g., flavonoids and alkaloids) act as potent modulators of cytochrome P450 (CYP) enzymes (such as CYP3A4, CYP2C9) and drug transporters (e.g., P-glycoprotein). Concomitant administration of TCM with cytotoxic chemotherapeutics or small-molecule targeted agents may unpredictably alter systemic drug exposure, increasing the risk of unexpected systemic toxicity or subtherapeutic antitumor efficacy.

Immunological Tensions with ICIs: Theoretically, the broad anti-inflammatory and immunosuppressive properties of certain TCM herbs—while beneficial for blunting systemic cachectic inflammation—carry a risk of dampening ICI-driven antitumor immunity. For instance, excessive inhibition of pro-inflammatory cascades necessary for acute T-cell priming could inadvertently compromise the therapeutic response to anti-PD-1/PD-L1 antibodies.

This theoretical tension is concretely illustrated by the dual role of TGF-β signaling in the context of ICI therapy and muscle toxicity. TGF-β is a genuinely double-edged mediator: in the tumor microenvironment, it suppresses CD8+ T cell cytotoxicity (repressing perforin, granzyme A/B, and IFN-γ), induces FOXP3+ Tregs and MDSCs, and polarizes macrophages toward immunosuppressive phenotypes—all of which contribute to primary resistance to anti-PD-1/PD-L1 therapy (149). Conversely, in skeletal muscle and heart, TGF-β predominantly exerts tolerogenic and anti-inflammatory effects, restraining CD8+ effector responses and protecting tissues from immunopathology (149). This compartment-specific dichotomy creates a concrete therapeutic paradox: dampening TGF-β-associated inflammation might mitigate ICI-induced myositis or myocarditis, but broadly suppressing inflammation or inadvertently augmenting TGF-β signaling in the tumor microenvironment could plausibly blunt ICI-driven anti-tumor immunity. While no direct evidence currently establishes TGF-β as the causal driver of ICI-induced myositis or myocarditis specifically—current mechanistic models center on autoreactive CD8+ T cells against α-myosin, CXCL9/10+ macrophages, IFN-γ, and the IL-6 axis (150)—the TGF-β double-edged sword construct highlights a general principle relevant to TCM adjuvants: any broadly immunomodulatory intervention has the theoretical capacity to shift the tumor-versus-toxicity balance in either direction. This underscores the critical importance of treatment timing, dosing, and patient selection—emphasized earlier as the “window of opportunity” paradigm—rather than administering TCM as an unmonitored co-therapy. Preclinical evidence suggests that when appropriately timed and combined with ICIs, certain TCM interventions may preferentially target the immunosuppressive TGF-β axis within the TME without broadly compromising systemic anti-tumor immunity, but this remains a hypothesis requiring direct investigation.

Therefore, comprehensive therapeutic drug monitoring, prospective toxicity reporting, and dedicated HDI screening are imperative before integrating TCM interventions into standard immunotherapeutic regimens.

### 5.4 The window of opportunity and critical appraisal of current evidence

Beyond the specific toxicity management strategies discussed above, the clinical positioning of TCM in cachexia care also depends on the optimal timing of intervention. According to the consensus framework endorsed by the EPCRC, ASCO, and ESMO, cancer cachexia is a three-stage continuum: pre-cachexia, cachexia, and refractory cachexia (6, 151). Refractory cachexia is clinically defined by progressive disease, an ECOG performance status of 3–4, and an expected survival of less than 3 months. At this terminal stage, the catabolic cascade—driven by irreversible mitochondrial failure, ubiquitous transcription of atrogenes, and terminal immune exhaustion—becomes largely unresponsive to both nutritional and pharmacological interventions.

The core clinical positioning of TCM is therefore consistent with the principle of “Zhi Wei Bing” (preventive treatment of disease)—supporting intervention during the pre-cachexia or early cachexia stages. By applying network-modulating formulas before the systemic inflammatory response and muscle ferroptosis reach an irreversible state, it may be possible to interrupt the pathogenic feedback loops that drive cachexia progression.

Current clinical data illustrate both the potential and the methodological limitations of this approach. Large-scale, real-world cohort studies utilizing the Taiwanese National Health Insurance Research Database have demonstrated that integrating TCM into standard oncology care prolongs OS and reduces all-cause mortality (HR 0.69) in malnourished cancer patients (152). Japanese Kampo medicines, including Rikkunshito and Hochuekkito, have shown evidence of benefit in mitigating anorexia and lowering serum IL-6 in retrospective and small prospective cohorts (153).

However, a rigorous appraisal identifies several limitations. The majority of these studies are limited by open-label designs, small sample sizes, and retrospective biases, including healthy-user bias. There remains a lack of large-scale, multi-center randomized controlled trials (RCTs) utilizing composite endpoints—the simultaneous assessment of survival, validated Quality of Life (QoL) metrics, and objective physical function (e.g., lean body mass plus 6-minute walk test)—as specified by regulatory bodies including the FDA for cachexia drug approval (154).

While conventional multimodal cachexia trials in Western oncology—such as the Korean MIRACLE trial, which integrated NSAIDs, omega-3 fatty acids, exercise, and nutritional support—have highlighted the challenges of trial accrual and endpoint achievement in advanced cancer populations (155). The challenges encountered in the MIRACLE trial—including difficulties in patient accrual, adherence to multimodal interventions, and the selection of meaningful endpoints—are emblematic of broader obstacles facing cachexia research. The MENAC trial, for example, required nearly seven years (May 2015 to February 2022) to randomize 212 patients, illustrating the difficulty of recruiting and retaining participants in advanced cancer populations (156). MIRACLE itself failed to reach its target sample size of 112 patients, directly reducing statistical power to detect differences in its primary endpoints (155). High attrition rates are intrinsic to progressive disease; the ASCO systematic review flagged dropout rates exceeding 50% in some multimodal RCTs, and inadequate capture of exercise adherence and nutritional intake may have masked potential benefits (6). Endpoint selection presents another critical hurdle: there remains no universally accepted primary endpoint for cachexia trials, and regulators have rejected agents such as anamorelin and enobosarm because lean mass gains were not matched by improvements in physical function—prompting a shift toward composite endpoints combining body weight with patient-reported outcomes (157). A recent Cochrane systematic review concluded that high-quality multimodal trials remain “in their infancy,” with heterogeneous inclusion criteria, small sample sizes, and high risk of bias across most studies (158). These same challenges apply to TCM-based trials, compounded by additional complexities such as batch-to-batch variability of herbal extracts and the need for culturally appropriate placebo controls. Overcoming these obstacles will require adaptive trial designs, patient stratification using standardized diagnostic criteria (e.g., GLIM), and the integration of validated translational biomarkers to identify treatment responders (2).

Analogous rigorous RCTs evaluating TCM-based interventions have not yet been conducted. This evidence gap highlights the urgent need for well-powered, multicenter, double-blind RCTs. To meet modern regulatory approval standards (e.g., US FDA and EMA guidelines for cachexia therapeutics), future trials must move beyond surrogate or single-metric outcomes and adopt regulatory-grade composite endpoints. Specifically, a gold-standard RCT protocol evaluating TCM adjuvants should concurrently integrate:

Hard Survival Outcomes: OS or progression-free survival under standardized ICI regimens;

Objective Physical and Nutritional Metrics: Quantitative changes in lean body mass (LBM, evaluated via CT scan/DEXA) combined with objective physical performance metrics (e.g., 6-minute walk distance test, handgrip strength); and

Validated Patient-Reported Outcomes (PROs): Health-related quality of life assessed through validated, cachexia-specific scales (e.g., EORTC QLQ-C30, FAACT anorexia-cachexia subscale).

Incorporating these composite endpoints alongside exploratory translational biomarkers (e.g., circulating cytokine panels, serum GDF15/LPS levels, and gut microbiome sequencing) is essential to yield unequivocal, high-level clinical evidence required by the global oncological community.

## 6 Conclusion and future directions

### 6.1 Summary

Cancer cachexia and concomitant tumor immune evasion are not parallel, independent syndromes; rather, they represent a self-amplifying disruption of systemic homeostasis (1, 4). As delineated throughout this review, these dual pathologies are linked by a shared molecular network: the aberrant crosstalk within the immunometabolic-ferroptosis-gut microbiota axis. Conventional single-target synthetic therapies have shown limited efficacy against this redundant, multi-organ pathogenesis. In this context, TCM—encompassing purified bioactive monomers and chromatographically standardized multi-herb formulas—represents a distinct therapeutic approach.

By functioning as multi-target network modulators, TCM interventions extend beyond the scope of conventional monotherapy. They produce a multi-level recalibration of host physiology. At the tissue level, these compounds inhibit skeletal muscle catabolism by suppressing the UPS and reducing lipid peroxidation to limit muscle ferroptosis. Concurrently, at the microenvironmental level, they counteract the immunosuppressive features of the TIME by downregulating PD-L1, inhibiting NLRP3 inflammasome assembly, and reversing M2 macrophage polarization via STAT3/NF-κB modulation. At the systemic level, TCM formulas remodel the gut microbiota, utilizing microbial metabolites such as SCFAs to repair the intestinal barrier, reduce endotoxemia, and epigenetically restore CD8+ T cell function.

This multi-dimensional pharmacological profile positions TCM as more than a supportive measure, offering systemic intervention across the interconnected pathologies of cachexia and immune evasion—an integrated strategy to disrupt the feedback loops that drive advanced cancer mortality.

### 6.2 Current limitations

Despite the mechanistic rationale supporting TCM as a systemic regulator of the cachexia-immunosuppression axis, translation from preclinical models to clinical practice is constrained by three methodological limitations.

#### 6.2.1 Preclinical confinement and pharmacokinetic properties of monomers

Monomer research remains confined to *in vitro* systems and preclinical rodent models. While natural compounds such as curcumin, EGCG, and astragaloside IV exhibit multi-target precision in laboratory settings, their clinical translation is limited by low aqueous solubility, rapid first-pass gastrointestinal metabolism, and limited systemic bioavailability. Whether these purified compounds reach pharmacologically active concentrations in human malignant tissues and dystrophic skeletal muscle at clinically feasible doses remains uncertain and requires further investigation.

#### 6.2.2 Incomplete characterization of multi-herb formulas

Despite advances in mass spectrometry fingerprinting that have improved the characterization of classical multi-herb formulas, their *in vivo* pharmacokinetic and pharmacodynamic (PK/PD) networks remain incompletely defined. Current literature is dominated by in silico network pharmacology predictions that frequently identify overlapping hub genes (e.g., AKT1, IL-6, TNF) across different formulas and disease contexts. There is a lack of system-level experimental verification capable of tracing the complete component–target–pathway–effect continuum *in vivo*, leaving the synergistic mechanisms of complex phytochemical matrices unresolved.

#### 6.2.3 Limited high-level clinical evidence

The clinical evidence base for TCM interventions in cancer cachexia is at an early stage and fragmented. Existing clinical data are predominantly derived from small-sample, single-center, retrospective, or open-label studies that are susceptible to unmeasured confounding and healthy-user bias. The field lacks high-quality, multicenter RCTs that utilize internationally accepted, regulatory-grade assessment criteria for cancer cachexia, leaving TCM without the high-level evidence required for integration into routine oncological practice.

### 6.3 Future directions

To address the limitations outlined above and to establish a rigorous scientific foundation for future investigations, research on TCM and cachexia should advance along three directions.

#### 6.3.1 Methodological innovation: spatiotemporal multi-omics mapping

To address the incomplete characterization of formula pharmacology, future research should move beyond bulk tissue analyses and in silico predictions toward spatiotemporal systems biology. The application of single-cell RNA sequencing (scRNA-seq) combined with spatial transcriptomics and spatial metabolomics may enable the generation of high-resolution cellular atlases of the muscle–adipose–tumor–gut multi-organ crosstalk under TCM intervention, defining how complex formulas alter the landscape of T cell exhaustion, macrophage repolarization, and cell-specific ferroptosis susceptibility at single-cell resolution.

#### 6.3.2 Molecular stratification: precision syndrome differentiation

The TCM principle of Bian Zheng Lun Zhi (syndrome differentiation and treatment) can inform the contemporary paradigm of precision medicine. Rather than approaching cancer cachexia as a single disease category, future investigations may apply molecular phenotyping to stratify patients into distinct endotypes—such as high-inflammatory (IL-6/TNF-α-driven), hyper-catabolic/nutritionally-depleted, or iron-dysregulated/ferroptotic subtypes. This stratification may enable the targeted evaluation of specific TCM formulas or monomers, matching a given intervention to the predominant pathological features of an individual patient.

#### 6.3.3 Conceptual innovation: integrating TCM compatibility principles into translational frameworks

Beyond molecular stratification, the holistic principles of TCM formula design—exemplified by the “Jun-Chen-Zuo-Shi” framework—offer a complementary paradigm for contextualizing multi-herb interventions in cachexia. This classical principle conceptualizes herbal formulas as hierarchically organized therapeutic teams, in which the “monarch” (Jun) herb provides the primary therapeutic effect, the “minister” (Chen) herbs enhance or support this effect, the “assistant” (Zuo) herbs address secondary symptoms or reduce toxicity, and the “guide” (Shi) herbs target the formula to specific organs or harmonize the actions of other components. While the framework has been explicitly applied in network pharmacology studies of anticancer formulas (e.g., breast cancer) (159) and quantitatively decoded in multi-scale systems pharmacology models (160), its direct application to cancer cachexia research remains limited. However, the same hierarchical logic—a principal herb supported by assisting herbs to achieve multi-target synergy while mitigating toxicity—is implicitly operationalized in several cachexia-relevant formula studies (65). The largest meta-analysis of herbal medicines for cancer cachexia (66 studies, 3,654 patients) reported improvements in KPS, weight, appetite, and quality of life, with recurring herb combinations (e.g., Ginseng, Astragalus, Atractylodes, Poria) that reflect an implicit monarch–minister structure (161). We therefore propose that the Jun-Chen-Zuo-Shi framework, while not yet validated as a standalone scientific model in cachexia, provides a valuable heuristic for designing and interpreting future network-modulating TCM interventions in this setting.

#### 6.3.4 Clinical trial design: composite endpoints with exploratory biomarkers

Clinical validation of TCM requires advances in trial design. Future trials should include large-scale, multicenter, double-blind RCTs with internationally validated composite endpoints. Primary outcomes may evaluate body weight stabilization, LBM measured by CT imaging, appetite improvement, and cachexia-specific quality of life assessed by standardized instruments (e.g., EORTC QLQ-C30, FAACT). These trials should also incorporate translational correlates as pre-specified exploratory endpoints—including immune cytokine profiles, T cell subpopulation dynamics, iron metabolism indices, and gut microbiome composition—to provide high-level evidence of TCM efficacy to the broader scientific community.
